# A protocol for a turbidimetric assay using a *Saccharomyces cerevisiae* thiamin biosynthesis mutant to estimate total vitamin B_1_ content in plant tissue samples

**DOI:** 10.1186/s13007-023-01117-8

**Published:** 2023-12-13

**Authors:** Simon Strobbe, Jana Verstraete, Teresa B. Fitzpatrick, Christophe Stove, Dominique Van Der Straeten

**Affiliations:** 1 Laboratory of Functional Plant Biology, Department of Biology, Ghent University, K.L, Ledeganckstraat 35, 9000 Ghent, Belgium; 2 Laboratory of Toxicology, Department of Bioanalysis, Ghent University, Ottergemsesteenweg 460, 9000 Ghent, Belgium; 3 Vitamins and Environmental Stress Responses in Plants, Department of Botany and Plant Biology, University of Geneva, Quai E. Ansermet 30, 1211 Geneva, Switzerland

**Keywords:** Turbidimetry, Microbiological assay, Thiamine, Protocol, Vitamin quantification, Biofortification, Metabolic engineering, Nutritional value, Nutrition, Plant breeding

## Abstract

**Background:**

Understanding thiamin (thiamine; vitamin B_1_) metabolism in plants is crucial, as it impacts plant nutritional value as well as stress tolerance. Studies aimed at elucidating novel aspects of thiamin in plants rely on adequate assessment of thiamin content. Mass spectrometry-based methods provide reliable quantification of thiamin as well as closely related biomolecules. However, these techniques require expensive equipment and expertise. Microbiological turbidimetric assays can evaluate the presence of thiamin in a given sample, only requiring low-cost, standard lab equipment. Although these microbiological assays do not reach the accuracy provided by mass spectrometry-based methods, the ease with which they can be deployed in an inexpensive and high-throughput manner, makes them a favorable method in many circumstances. However, the thiamin research field could benefit from a detailed step-by-step protocol to perform such assays as well as a further assessment of its potential and limitations.

**Results:**

Here, we show that the *Saccharomyces cerevisiae* thiamin biosynthesis mutant *thi6* is an ideal candidate to be implemented in a turbidimetric assay aimed at assessing the content of thiamin and its phosphorylated equivalents (total vitamer B_1_). An optimized protocol was generated, adapted from a previously established microbiological assay using the *thi4* mutant. A step-by-step guidance for this protocol is presented. Furthermore, the applicability of the assay is illustrated by assessment of different samples, including plant as well as non-plant materials. In doing so, our work provides an extension of the applicability of the microbiological assay on top of providing important considerations upon implementing the protocol.

**Conclusions:**

An inexpensive, user-friendly protocol, including step-by-step guidance, which allows adequate estimation of vitamer B_1_ content of samples, is provided. The method is well-suited to screen materials to identify altered vitamer B_1_ content, such as in metabolic engineering or screening of germplasm.

## Background

Thiamin metabolism is essential in all life forms, as it is required to supply a cofactor in multiple crucial reactions in central energy metabolism [6, 13]. Thiamin and its phosphorylated entities, thiamin monophosphate (TMP), thiamin pyrophosphate (TPP) and thiamin triphosphate (TTP) [10] are collectively referred to as B_1_ vitamers (vitB_1_). These B1 vitamers are chemical derivatives that can be interconverted within the human body. Therefore, adequate metabolic function of our body, which requires TPP, relies on a form of B_1_ vitamer. Humans are unable to biosynthesize thiamin de novo, and are thus almost exclusively relying on their diet for sufficient acquisition of vitB_1_, as the gut microbiome only supplies a fraction of the vitB_1_ required [14]. The recommended daily allowance (RDA) for vitB_1_ is set at 1.2 mg for adult men and women and 1.4 mg for pregnant women [32]. Severe or chronic vitB_1_ insufficiency is known to have a devastating impact on human health, as the effects include cognitive impairment and cardiovascular pathologies [38]. Ensuring satisfactory thiamin intake in different populations can be enforced via dietary diversification, supplementation, food processing and biofortification [39]. The latter involves the enhancement of natural vitB_1_ levels in crops via breeding, genetic engineering or agronomic interventions [7]. In this respect, metabolic engineering of vitB_1_ content has been undertaken in the crop species rice (*Oryza sativa*) [4, 29] as well as in the model plant *Arabidopsis thaliana* [3, 30]. While metabolic engineering via genetic engineering primarily relies on adequate knowledge of plant vitB_1_ metabolism [7, 11, 22, 24, 26, 27], breeding is more dependent on sufficient variability in vitB_1_ content in the available sexually compatible germplasm of the particular crop of interest [9, 15, 16, 34]. Both these interventions, however, are reliant on adequate assessment of vitB_1_ content in the edible tissues of the crop of interest. Quantification of vitB_1_ in plant samples can be achieved using high-performance liquid chromatography (HPLC) or mass spectrometry (MS)-based methodologies [18, 35]. Unfortunately, these techniques require expensive, specialized equipment as well as skilled operators. Easily applicable, inexpensive methods for vitB_1_ quantification can be very valuable, having an application potential beyond the aforementioned plant improvement, as they could also serve to investigate variations in vitB_1_ content of a wide range of samples, such as different plant materials as well as animal-derived products and even food supplements. Historically, multiple methods of thiamin quantification have been utilized, including microbiological assays as well as fluorescence-based methods [5]. The latter includes the thiochrome assay, which quantifies blue fluorescent thiochrome that is formed after the oxidation of thiamin. Thiochrome assays are widely used but are known to exhibit interference from antioxidant products such as ascorbate (vitamin C, ascorbic acid) and polyphenols [5]. Microbiological assays have proven their usefulness to quantify thiamin in biological samples [2, 5, 19] and have more recently been shown to be a fast and low-cost alternative to HPLC or MS-based methods [8, 15, 16, 24, 28]. A variety of organisms have been used in these turbidimetric assays, including *Streptococcus salivarius* [19] and *Lactobacillus viridescens* [1, 2, 8, 9] and *Saccharomyces cerevisiae* [15, 16, 24, 28]. These assays are predominantly turbidimetric, exploiting vitB_1_ dependent growth of microorganisms. The methodology makes use of mutant strains, wherein a specific step in vitB_1_ biosynthesis is affected. In those cases, depending on the exact step of the biosynthesis that was omitted, several other thiamin-related metabolites could be interfering with the microbiological assay [19, 28]. Indeed, pyrimidine and thiazole metabolites, present as metabolic intermediates in vitB_1_ biosynthesis as well as occurring as breakdown products, have been reported to interfere with the measurement of vitB_1_ microbiological assays [5, 19, 28]. This highlights the necessity of selecting ideally suited mutant lines to allow a correct estimation of the desired metabolites. Recently, we have exploited the available genetic resources in *Saccharomyces cerevisiae*, by examining different strains mutated in vitB_1_ biosynthesis, to assess the applicability of different strains to determine vitB_1_ content [28]. This revealed that the *Saccharomyces cerevisiae* strain *thi6,* which is unable to condense pyrimidine and thiazole intermediates to form TMP (the first vitB_1_ vitamer in vitB_1_ biosynthesis) [20, 21]*,* is the strain of choice to be deployed in a microbiological assay determining vitB_1_ [28]. Interestingly, its combined use with the RWY16 strain [40] and the previously described assay utilizing the *thi4* strain [15, 24], enabled rough estimation of pyrimidine and thiazole content in plant samples, respectively [28]. This nicely illustrates how a panel of different vitB_1_ mutant strains within the same organism can be addressed to permit a more profound insight into metabolites involved in vitB_1_ metabolism, via their implementation in microbiological assays. Furthermore, *Saccharomyces cerevisiae* is an ideal tool, as it is a model organism, which is considered safe and can be grown fast in standardized growth conditions.

Here, we demonstrate the applicability of the *Saccharomyces cerevisiae* strain *thi6* in a microbiological assay aimed at estimating vitB_1_ content and provide step-by-step guidance (in the form of a lab protocol). Important considerations regarding the execution and reproducibility of the assay are examined and elaborately discussed. Finally, we illustrate the application of the assay by screening rice samples as well as a more varied set of plant- and non-plant-derived samples. Together, our work presents support for the wide applicability of the assay and provides the necessary information to allow user-friendly implementation of the methodology.

## Results

### Timing of thiamin dependent growth in *thi6* strain and its response to different vitamers

**Fig. 1:**
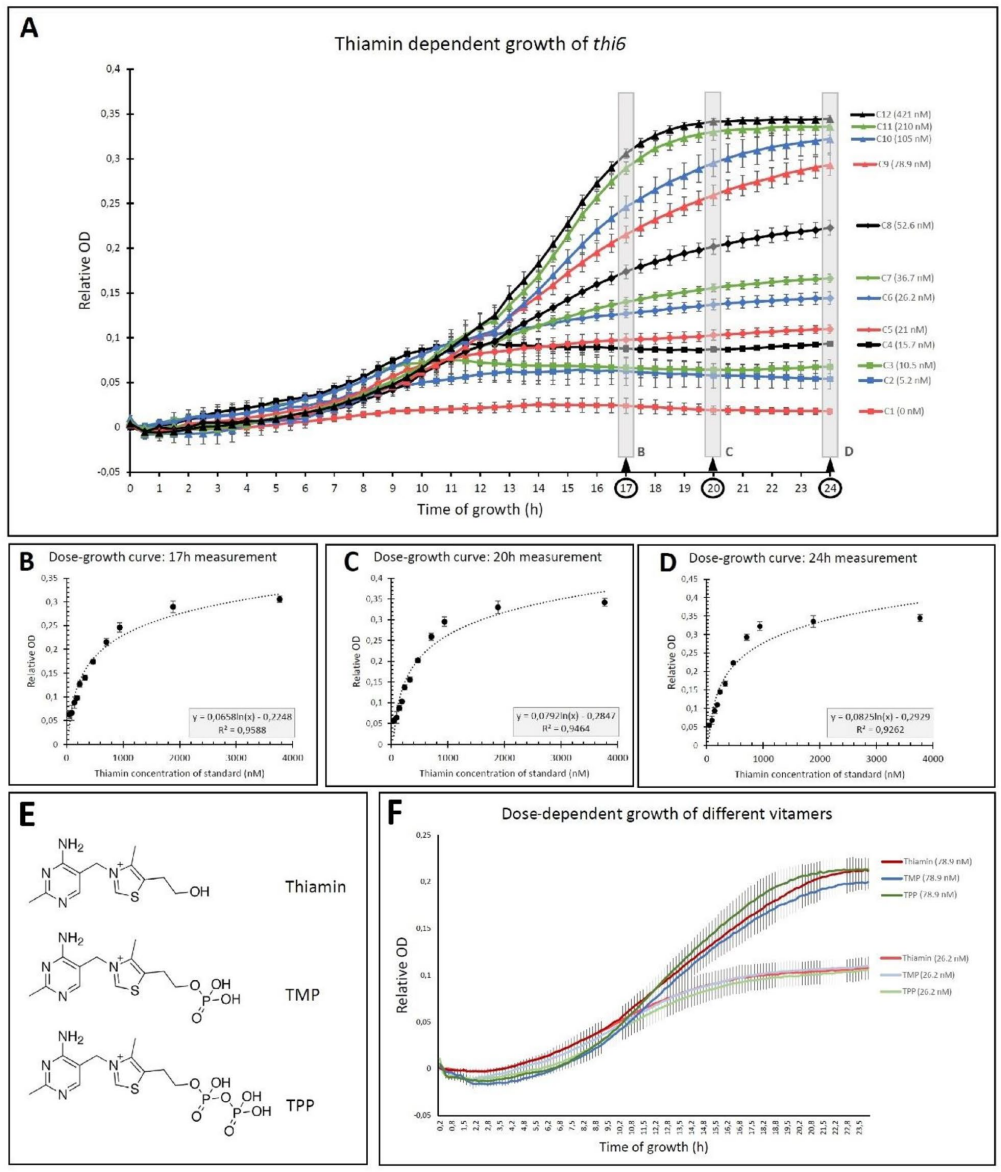
Timing of *thi6* thiamin concentration-responsive growth. The *thi6 Saccharomyces cerevisiae* mutant strain depicts a thiamin concentration-responsive growth when grown on different concentrations of thiamin standards [28]. **A** Quantification of the growth of *thi6* was monitored in real-time as a proxy for thiamin content (see methods) using a TECAN Infinite 200 pro plate reader. The graph depicts the *thi6* growth progression by monitoring Optical Density (OD), for twelve different thiamin concentrations, at 30 min timepoint intervals, for a total period of 24 h (h). Each concentration of the thiamin standard was tested in two technical replicates (2 × 12; 24 well plate). The data represent the mean ± Standard Error of at least 7 repeats (7 different days of measurements). Gray rectangles indicate timepoints that were used to generate growth concentration–response curves of the *thi6* strain in the microbiological assay. Concentration-dependent growth curves were assessed at 17 h (**B**), 20 h (**C**) and 24 h (**D**) of growth. The correlation (R^2^) between the concentration of thiamin standard added, and the OD for a given timepoint of measurement is indicated. Taking the example of the 17 h timepoint, the concentration of thiamin in the standard is estimated by the following formula: concentration (nM) = e^(OD+0.2248)/0.0658^. Note that the concentrations depicted (ranging from 0–421 nM) arise from the thiamin standard dilution series (ranging from 0–3770 nM) as used in the assay protocol (see methods for the preparation of the thiamin standard series and assay). **E** Chemical structure of most common vitamin B_1_ vitamers. **F** Concentration-dependent growth of *thi6* strain using two distinct concentrations of the most common vitamin B_1_ vitamers (mean ± Standard Error of 3 repeats (analysis days) each consisting of 2 technical repeats, are shown). OD values were derived from the TECAN Infinite 200 pro plate reader, in which the measurements represent the measured OD subtracted by the OD measured at timepoint 0. Moreover, as the reading does not include correction for path length, OD values should only be interpreted in a relative way. *TMP* thiamin monophosphate, *TPP* thiamin pyrophosphate

It was recently shown that the *Saccharomyces cerevisiae* strain *thi6* can serve in a vitB_1_ turbidimetric assay, as it is responsive to thiamin, TMP and TPP in approximately equal molar quantities [28], which is in line with the current understanding of yeast vitB_1_ metabolism and the positioning of the omitted step, executed by THI6, within the pathway [20, 21]. To confirm the vitB_1_-dependent growth of this strain, as well as to obtain a deeper insight into the timing of its growth, the *thi6* strain was examined at varying vitB_1_ concentrations (Fig. 1). This is relevant to optimize the turbidimetric assay with respect to the time needed during the growth phase on the one hand and to get an idea of the severity of impact of deviating from this timing on the other hand. The results show that after 8 h of growth, a clear distinction was observed between the *thi6* culture grown in thiamin-deprived conditions (C1; 0 nM control) as compared to thiamin-supplemented conditions (Fig. 1A). It is important to note that, in certain cases, some aberrant growth was observed, reflecting the highest growth during the early stages of the assay (e.g. C4 at 8 h, Fig. 1A). This can potentially be caused by impurities or condensate formation on the 24-well plate, also indicating that attention should be paid to the proper reading of optical densities, which should be done after longer periods of growth. The 17, 20 and 24 h timepoint were selected to assess the thiamin growth concentration–response curve, for which the 17 h timepoint most closely correlated (R^2^ > 0.95) to the logarithmic mathematical approximation of the growth-concentration–response curve (Fig. 1B–D). Analysis of the correlation between measured optical densities (OD) and thiamin concentration provides an idea of the optimal time of growth for measurements, displaying the highest correlation. Focusing on the optimal concentration (i.e. excluding the 2 lowest and 2 highest concentrations) revealed that the highest correlations of OD and thiamin concentration were indeed obtained at the start of the plateau phase of growth, after 16, 17 and 18 h of growth, with an R^2^ value of 0.98 (14 h, R^2^ = 0.951; 15 h, R^2^ = 0.972; 16 h, R^2^ = 0.979; 17 h, R^2^ = 0.981; 18 h, R^2^ = 0.979; 19 h, R^2^ = 0.971). Interestingly, the results show that suboptimal supplementation of thiamin (e.g. C7; 36.7 nM; Fig. 1A) not only slows *thi6* growth, but also results in lowered plateau of OD reached. The results indicate that a ≥ 17 h growth time allows adequate vitB_1_ estimation. The three thiamin related B_1_ vitamers most commonly found in biological samples, depicted in Fig. 1E, had an equal effect on the growth of the thi6 strain, when provided in the same molar concentrations (Fig. 1F), confirming similar detectability of the different phosphorylated entities as compared to thiamin itself [28]. Indeed, testing different mixtures (33.3% each, 50:25:25, 25:25:50) of these three vitamers demonstrated that growth of *thi6* was dependent on the molar concentration of total vitB_1_ (sum of the different vitamers) in the assay media (data not shown).

### Other nutritional components as well as the age of the yeast stock can impact growth

**Fig. 2:**
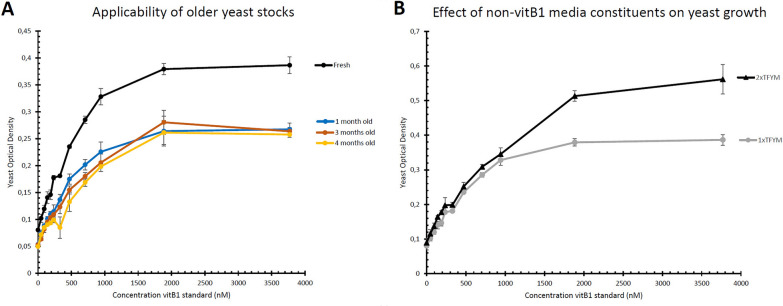
Factors influencing yeast growth during assays. The thiamin concentration-responsive growth of the *thi6 Saccharomyces cerevisiae* mutant strain, used for turbidimetric assay of vitB_1_ quantification [28], was examined upon usage of fresh or stored yeast cultures (**A**) or higher nutrient composition of the TFYM growth medium (**B**). Following the assay protocol, Optical Density (OD) was measured after 17 h of growth. **A** Here, however, small adaptations to the protocol were tested, as stored, washed yeast cultures of different age (stored at 4 °C for 1, 3 or 4 months) were utilized to test their thiamin concentration-responsiveness. Note that the cultures were tested at a similar time, meaning that these originated from different *thi6* cultures separately washed (not one culture tested at different timepoints). **B** Similarly, *thi6* growth was followed upon growth in double concentration TFYM, to examine whether higher nutrient composition can have an effect on the assay outcome. Error bars represent standard deviation as measured from 4 technical replicates

Similar to investigating the timing of the growth, it is important to explore which other factors have an impact on the yeast growth, as this could potentially influence the outcome of the microbiological assay. Such insight provides the user with knowledge on how stringent certain factors should be controlled. Testing the ability of ‘older’ yeast stocks, being yeast cultures washed and stored at OD 0.5 at 4 °C to be used in the assay, would eliminate the necessity to prepare a fresh yeast culture for each assay day (see “Preparation of the yeast culture”). Our results show that using yeast cultures, stored for up to 4 months, still depict clear vitB_1_-responsive growth, albeit to a lower extent as compared to the fresh stock (Fig. 2A). Though this effect is much more clear for higher vitB_1_ concentrations, in the lower linear range of the curve (which is the recommended range of the assay [28]), adequate concentration-dependent growth is observed. Conversely, using a twofold higher concentration of growth media, the concentration-responsive growth is extended, as the *thi6* mutant grows to a higher OD (Fig. 2B). Similarly, this effect is limited in the earlier, linear, phase of the curve. This observation illustrates that certain components of the plant extracts, unintentionally co-extracted together with vitB_1_, could also influence the assay outcome.

### Reproducibility

**Fig. 3:**
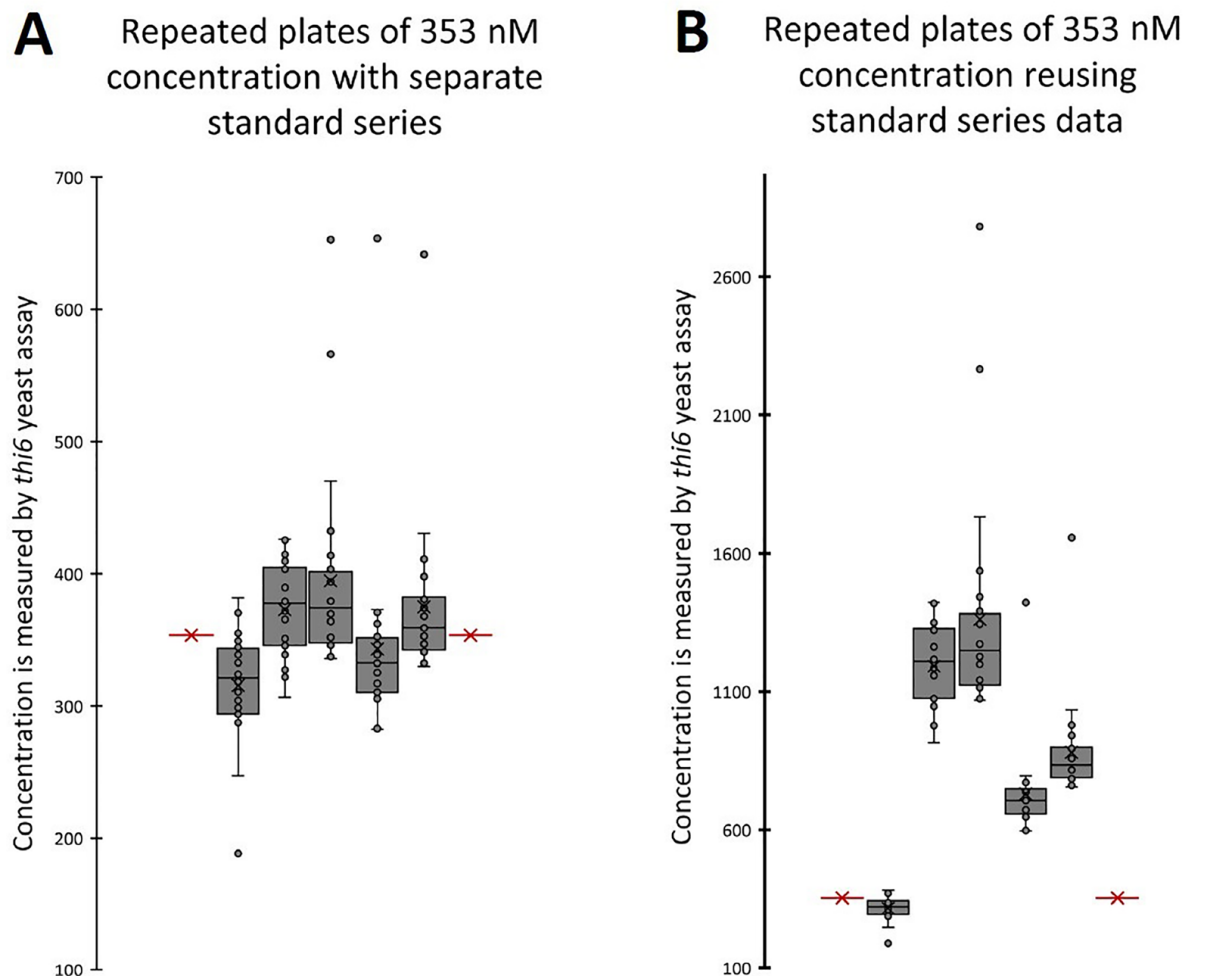
Standard series are required for reproducibility of the microbiological assay. The vitB_1_ *thi6* microbiological assay was used on a 353 nM thiamin aqueous test solution. Each analysis day (17 h assay incubation) consisted of a standard series (12 concentrations, 4 technical replicates, 48 wells in total) and 24 technical replicates of the 353 nM test solution (24-well plate). The box plots show the median and quartiles, where X represents the mean. Measurements, including outliers, are shown as points. The red crossed line indicates the expected, true, concentration of the test sample, 353 nM. Each result is calculated by using the concentration–response curve from the standard series, which is measured in parallel for each analysis day (**A**). Analyses in which no standard series are included for each day are presented in **B**. Here, the equation derived from the assay of the first analysis day (box plot on the left) was reused to calculate the test solution in the subsequent analysis days

The reliability of an assay depends on its accuracy and consistency. To estimate this, we conducted five microbiological assays on the same 353 nM thiamin test concentration. This concentration resides within the optimal measuring range and was prepared as a twofold dilution of the stock concentration C9 (see “Preparation of the thiamin standard series”). The measured mean values of the repeats were found to be within a range of 353 nm ± 12% for the 5 analysis days, provided that each assay was accompanied by a standard curve to derive the concentration–response relationship (Fig. 3A). To evaluate whether the latter is imperative, the concentration–response equation, derived from the first analysis day, was used (or reused) to estimate the concentration of the test solution of the other analysis days. Reiterative application of the concentration–response curve led to inaccurate estimation of the concentration and thus higher variation between different analysis days (Fig. 3B). Independent of whether or not the standard series were correctly included, outliers were found, demonstrating that high variation can occur for a limited number of replicates. Therefore, it is crucial to include adequate replicates to be able to identify these values as outliers, which could originate from errors in OD reading, condensate formation (on plate), coagulations of the yeast and impurities on the plate (when using a plate reader).

### Application of the *thi6* yeast assay on rice and other plant and non-plant samples

**Fig. 4:**
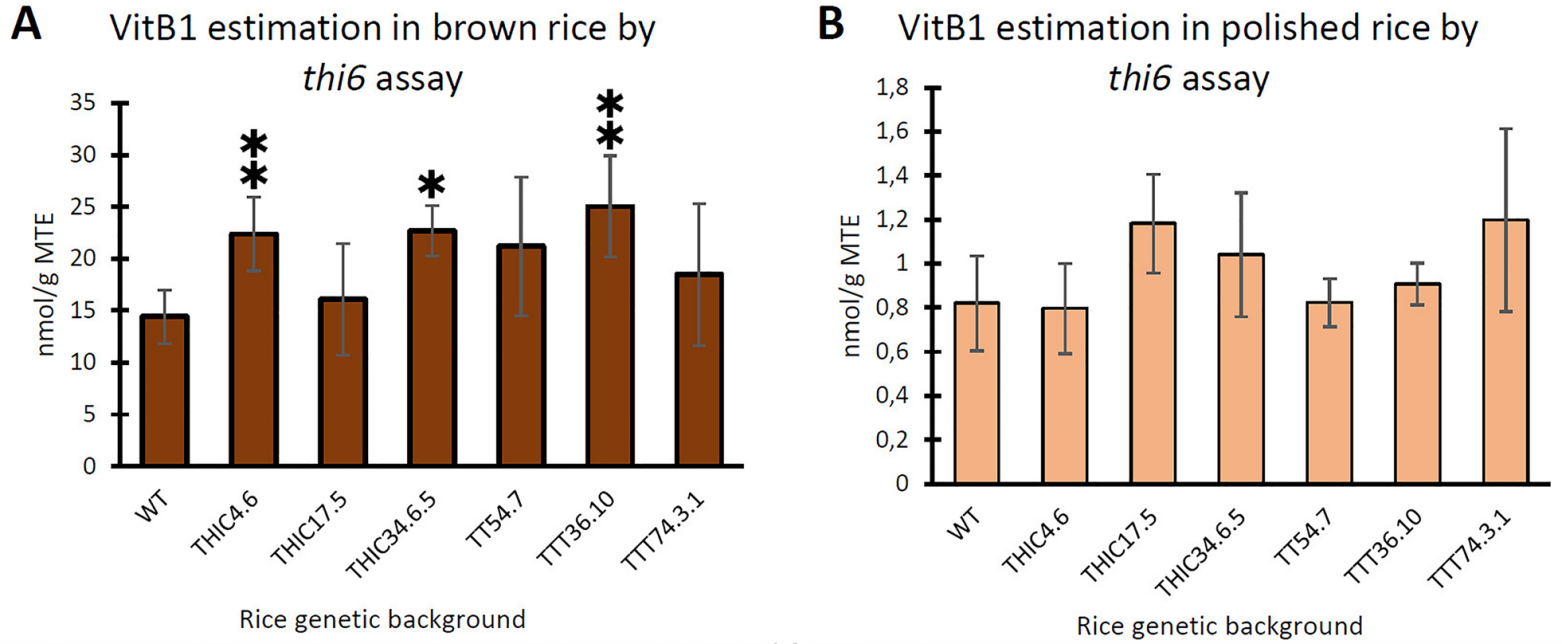
VitB_1_ estimation in different samples using *thi6* yeast microbiological assay. The *thi6* yeast assay, as described, was used to assess vitB_1_ content in different samples. An estimate of vitB_1_ content was obtained for brown (unpolished) (**A**) and polished (**B**) rice, including wild type (WT) and different genetic backgrounds generated in an earlier biofortification project via genetic engineering [29]. Values represent means ± standard deviation (SD) of 3 (WT, N = 6) biological repeats, each consisting of 2 technical replicates. Significant differences (p < 0.01; 2 asterisks; 0.01 < p < 0.05, 1 asterisk) were evaluated via a 2-sided Student’s *t*-test, of which the scedasticity was depending on the outcome of the F-test, as wild type values were found to depict normal distribution (Shapiro–Wilk test) allowing for parametric tests. Comparison of brown vs. polished wild type rice seeds revealed a significant (p < 0.001) drop in vitB_1_ upon polishing

To illustrate the applicability in relevant plant tissue samples depicting different vitB_1_ profiles, the *thi6* assay was used to examine the vitB_1_ content of both brown and polished transgenic *Nipponbare japonica* rice, with a large discrepancy in total vitB_1_ level [29]. Note that vitB_1_ concentrations, as estimated via the assay, are expressed as Molar Thiamin Equivalent (MTE), as the assay is unable to distinguish between the different vitB_1_ entities and relies on comparison to a thiamin standard series. Here, brown (unpolished) rice was estimated to contain about 14.4 nmol/g MTE or 383 µg/100 g thiamin (Fig. 4a), which is in the same order of magnitude as 413 µg/100 g reported by the USDA Food Data Central [33] as well as the 295 µg/100 g found earlier via MS-based quantification [29, 36]. The assay was able to identify significant differences, as compared to the wild type, in brown rice. In polished white rice on the other hand, no significant differences were found, although all polished samples were observed to significantly differ from the unpolished ones (p < 0.05) (Fig. 4B). Clearly, the assay can be applied to examine samples with a large variety in vitB_1_ content, as brown rice was found to contain above 15-fold more vitB_1_ as compared to polished white rice. This example also illustrates that, although measured variation can be high in low vitB_1_ samples, the assay can provide an estimate on losses during practices such as polishing.

**Fig. 5:**
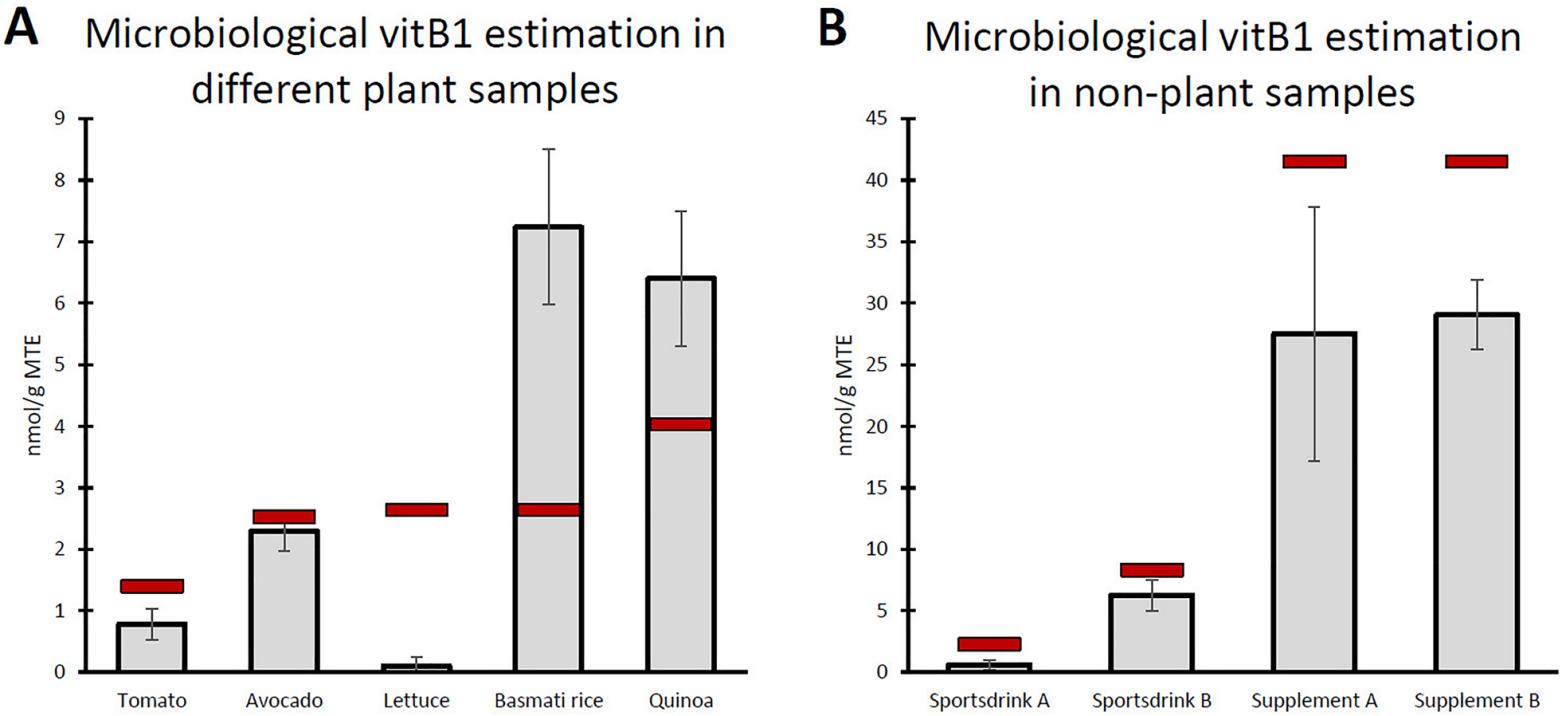
VitB_1_ estimation in plant as well as non-plant samples. The *thi6* yeast assay, as described, was used to assess vitB_1_ content in plant (**A**) and non-plant (**B**) samples. Values represent means ± standard deviation (SD) of 3 biological repeats (**A**) or technical (**B**) replicates. The plant materials (**A**) consisted of the edible tissues of the included crops (tomato and avocado, fresh fruit tissue; lettuce, fresh leaves, basmati rice and quinoa, complete seeds). Non-plant materials (**B**) consisted of 2 liquid sports drinks and 2 solid vitamin supplements. The latter were first dissolved and homogenized in water, after which all samples were sterilized and included in the yeast assay, similar to the plant extracts. Red bars indicate expected vitB_1_ content based on reported thiamin content on the USDA Food Database [33] for the plant material (with the exception of basmati rice for which data from Sood and colleagues [25] was used) or the manufacturer’s reported thiamin content for the non-plant materials

Furthermore, the assay was used to estimate vitB_1_ content in different plant tissues as well as potential samples of interest from non-plant origin. Fresh edible portions of a few crop plants, including tomato, avocado, lettuce, basmati rice and quinoa were examined (Fig. 5A). It is noteworthy that the basmati rice samples (*Indica*, long grain), though exceeding its expected vitB_1_ content, had only 50% of the vitB_1_ content observed in *Nipponbare* (*Japonica*, short grain) (Fig. 4A). On top of plant-derived samples, liquid thiamin containing sports drinks as well as vitamin supplements, were examined as non-plant materials (Fig. 5B). The results demonstrated that the assay is able to quantify vitB_1_ content from this variety of samples, roughly corresponding to the expected thiamin content, with the exception of the lettuce samples. Note that the high thiamin levels in the supplements were quantified by making consecutive dilutions of the sample and using the data for which the measurements reside in the optimal early phase of yeast growth (see also point-by-point protocol “Preparation of plant tissue samples”). In doing so, this methodology allows for vitB_1_ estimation of samples with very high expected vitB_1_ content, such as supplements. This showcases how the assay can provide an insight into vitB_1_ content of many different types of materials in a relatively easy way.

## Discussion

The results illustrate the applicability of the *thi6 Saccharomyces cerevisiae* strain to determine, in a semi-quantitative (relative) way, vitB_1_ content and further reveal its versatility and robustness. The experiments provide an insight into the conditions to take into account when conducting the turbidimetric assay as well as in the types of samples that can be examined.

The observation that the *thi6* yeast strain displays a different plateau depending on the added concentration of thiamin (Fig. 1A) has interesting implications as it indicates that some variation is allowed in the actual timing of OD measurements. This flexibility in timing is useful, as some inevitable time lost between measurement of samples and standards will not have a detrimental impact on the measurement variation as the OD is entering a plateau phase in time (Fig. 1A). This becomes particularly interesting if no automated OD measurement is available. Conversely, limiting the required growth time aids in reducing the total time required to perform the analysis as well as limiting the potential harmful effect of contamination. This favors the 17 h timepoint, which also depicted the highest correlation efficient (R^2^: 0.9588) with the logarithmic description of the relation between OD and thiamin concentration. The thiamin dependent growth experiments (Fig. 1A) also revealed that growth on the lowest thiamin concentration (C2; 5.2 nM) was adequately distinguishable from growth in the absence of thiamin (C1; 0 nM). Therefore, a 5 nM concentration in the assay volume can be considered adequately detectable. Given that the sample is ninefold diluted in the assay mixture (see “Preparation of the assay”), this requires samples to contain approximately 50 nM vitB_1_ to be detectable. This sensitivity could likely be increased by increasing the amount of sample in the assay, from 10 to 50% of the assay volume (i.e. replacing the sterile water). In doing so, the limit of detection can be lowered to 10 nM of vitB_1_ in a sample.

By examining growth of yeast cultures stored for longer periods of time it was revealed that older yeast cultures can be used, but this could narrow the range and sensitivity of the assay (Fig. 2A). The observation that a higher concentration of growth media (double concentrated TFYM) significantly impacted the yeast growth (Fig. 2B) is a warning that other compounds, potentially included in the crude vitB_1_ extract, can also influence growth and hence the outcome of the assay. Moreover, some samples may contain growth inhibiting compounds, such as antifungal alkaloids [23]. This effect can be, at least partially, avoided by only comparing samples of similar content, having a similar matrix in which vitB_1_ was extracted (e.g. seeds from different rice lines; stored and non-stored supplements).

Some adaptations to make the assay less labor intensive, were explored. This includes the aforementioned use of older yeast cultures, as well as reusing previously analyzed standard series. The latter was shown to severely diminish the accuracy of the assay (Fig. 3B) and is, therefore, strongly discouraged. It is indeed tempting to reuse an obtained concentration–response curve for future analysis days, thereby saving the need for a specific standard series per analysis, which depending on the number of samples, can become a significant part of the measurements. The results in Fig. 3, however, emphasize the harm of omitting this step, as reusage of concentration–response equations greatly increases variation and has the potential to lead to inaccurate results. A standard should therefore only be used together with samples arising from the same assay master mix on a specific analysis day.

The assay has at least the possibility to provide relatively accurate measurements, shown by the limited variability witnessed in Fig. 3A. This should, however, be approached with caution, as this was demonstrated on an aqueous solution, with a high number of technical repeats (n = 24), representing optimal conditions. Taking factors such as sampling, weighing, extraction and handling errors into account, variability can greatly increase. Furthermore, it is important to keep in mind that the assay results should be interpreted relatively, and used to compare similar sample materials, so that factors such as growth inhibiting compounds and extraction efficiency are similar between different samples.

The results in Fig. 5 show that the assays can be deployed in a wide range of materials. It is important, however, to get an idea about the expected vitB_1_ concentration and dilute the sample to fit within the optimal range of the assay (see “Preparation of plant tissue samples”), as was done for the vitB_1_ supplements (Fig. 5B).

It is important to keep in mind that the assay is a very crude estimation and should be utilized in experiments in which such estimation is sufficient or within preliminary experiments, requiring verification via MS-based methods.

## Conclusions

The *thi6* yeast turbidimetric assay can be used to provide estimates of vitB_1_ content in different samples. However, this requires relatively fresh yeast stocks and a standard series included for each analysis day. The assay assesses vitB_1_ levels as a sum of the different vitamers, measured as molar thiamin equivalent (MTE), as it cannot distinguish between these different metabolites. The resulting measurements should be considered as relative estimates rather than absolute quantification and can be used for semi-quantitative purposes, as in screening of large germplasm populations. The provided step-by-step protocol and the accompanying considerations allow for easy implementation of the turbidimetric *thi6* assay.

## Methods

**Fig. 6:**
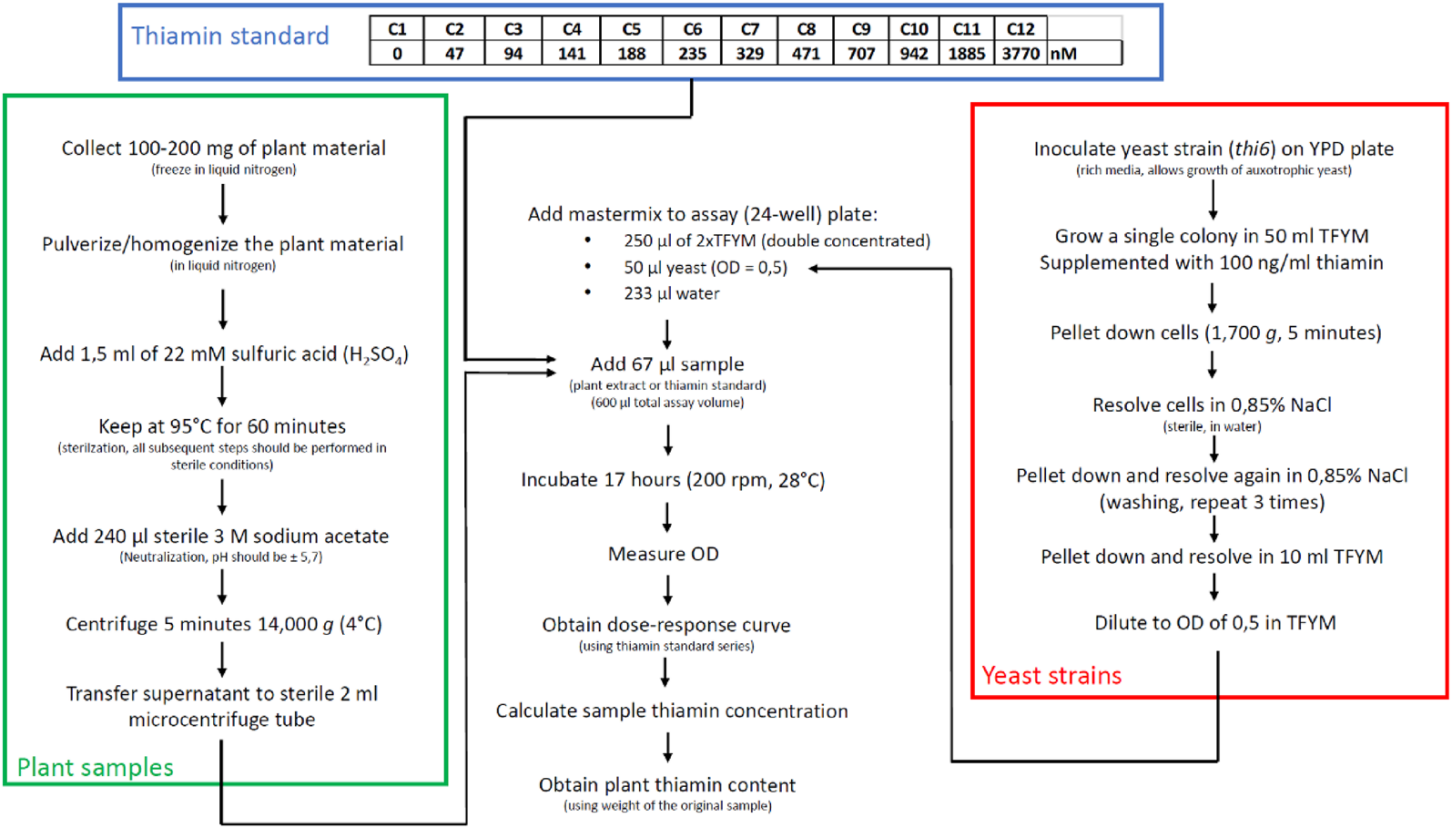
Flowchart of yeast microbiological assay for thiamin determination. The green frame represents the preparation of the plant samples. The blue frame shows the 12 concentrations (C1 to C12) of the thiamin standard in nM. The red frame covers the preparation of the yeast cultures. YPD, yeast extract-peptone-dextrose medium; TFYM, Thiamin-free yeast medium (ForMedium: CYN4701 + ForMedium: DCS0011 + 1% sucrose); OD, optical density

Herewith, an elaborate description of the procedures involved in the microbiological assay is provided. The methodology and its preparations can be subdivided in four parts, the preparation of the plant extracts, the thiamin standard dilution series, the preparation of the yeast culture and the combination of the aforementioned parts into the actual turbidimetric assay. In the following sections, these procedures will be reviewed and followed by a comprehensive step-by-step protocol. A flowchart of the complete procedure is presented in Fig. 6.

### Detailed protocol

#### Thiamin extraction

The extraction of vitB_1_ from plant material is based on previously described extraction methods for vitamin B6 [12, 17, 31], adapted for vitB_1_ [8, 15, 24].

To create a sample extract, as low as 100 to 200 mg of plant material can be used. The amount of material is chosen based on availability as well as on the expected vitB_1_ content, where 100 mg would be sufficient for high vitB_1_ sources (e.g. green plant tissues such as Arabidopsis leaves), while 200 mg is required for plant material with a low vitB_1_ content (e.g. polished rice seeds). The weight of each sample is accurately measured. Ideally, the sample material should be flash frozen in liquid nitrogen and stored frozen (preferentially at − 80 °C) until downstream processing. The material should be completely powdered to ensure efficient extraction. This can be done in an automated way (milling machine) or using mortar and pestle (both cooled using liquid nitrogen). In case the latter option is chosen, swift weighing of the plant material after homogenization will be required due to potential losses during handling. After homogenized samples are obtained, 1.5 ml of 22 mM of sulfuric acid (H_2_SO_4_) is added and the samples are heated to 95 °C for 60 min. This high temperature step assists in adequate extraction of vitB_1_ from the plant tissues while providing sterilization of the material, which is required as contamination could obstruct the assay, as described previously for vitB6 [12] and vitB_1_ [24]. Moreover, the low pH aids in limiting thiamin breakdown, as thiamin is very stable in acidic conditions [37]. All subsequent steps are performed in sterile conditions to avoid contamination of the sample extracts. To neutralize the solution to a pH of 5.7, 240 µl of sterile (autoclaved) 3 M sodium acetate (C_2_H_3_NaO_2_) is added to the sample. To eliminate the presence of cellular debris and other impurities, which could interfere with optical density (OD) measurements, extracts are centrifuged (14,000*g*; 4 °C) after which the supernatant is collected in a sterile microcentrifuge tube. This aqueous vitB_1_ extract is then directly used in the yeast assay (described below) or stored at − 80 °C for future analysis.

#### Thiamin standards

Thiamin hydrochloride (Duchefa Biochemie T0614; molecular weight (MW), 337.3 g/mol) is used to create a vitB_1_ dilution series in sterile distilled water, serving as a standard to deduce quantitative information in the yeast assays. Sterility of the thiamin stock solution is achieved via filter sterilization. Twelve different concentrations (ranging from 47 to 3770 nM, including a blank control) are included to obtain the concentration–response curves, which are used to deduce vitB_1_ content of the extracts (Fig. 6; described further).

#### Yeast culture

The *Saccharomyces cerevisiae* knockout mutant *thi6* (BY4741 background, MATa, YPL214C mutation) was purchased from the Euroscarf collection (EUROpean Saccharomyces Cerevisiae Archive for Functional analysis). Yeast extract-Peptone-Dextrose (YPD) medium is used to propagate and maintain the strain, as this medium contains a sufficient amount of vitB_1_. A cell culture of the *thi6* strain is grown, originating from a single colony on a YPD plate, in Thiamin-Free Yeast Medium (TFYM), supplemented with 100 ng/ml (377 nM) filter-sterilized thiamin (MW: 265.3). TFYM combines the yeast nitrogen base without amino acids and without thiamin (CYN4701, ForMedium), enriched with complete supplement medium (DCS0011, ForMedium) and 1% sucrose. Following the manufacturer’s instructions to make 1 L double concentrated TFYM (2 × TFYM), 13.8 g CYN4701, 1.58 mg DCS0011 and 20 g sucrose are dissolved in distilled water and autoclaved. TFYM supplemented with 377 nM thiamin is used to grow the *thi6* culture (OD > 1) overnight (28 °C, 200 rpm). Subsequently, the *thi6* culture is pelleted by centrifugation (1700*g*, 5 min), washed 3 times with sterile aqueous 0.85% NaCl solution (for elimination of residual thiamin) and re-dissolved in TFYM at an OD of 0.5. The diluted *thi6* culture solution (OD: 0.5) can be directly used in the assay or stored at 4 °C.

#### Assay

**Fig. 7:**
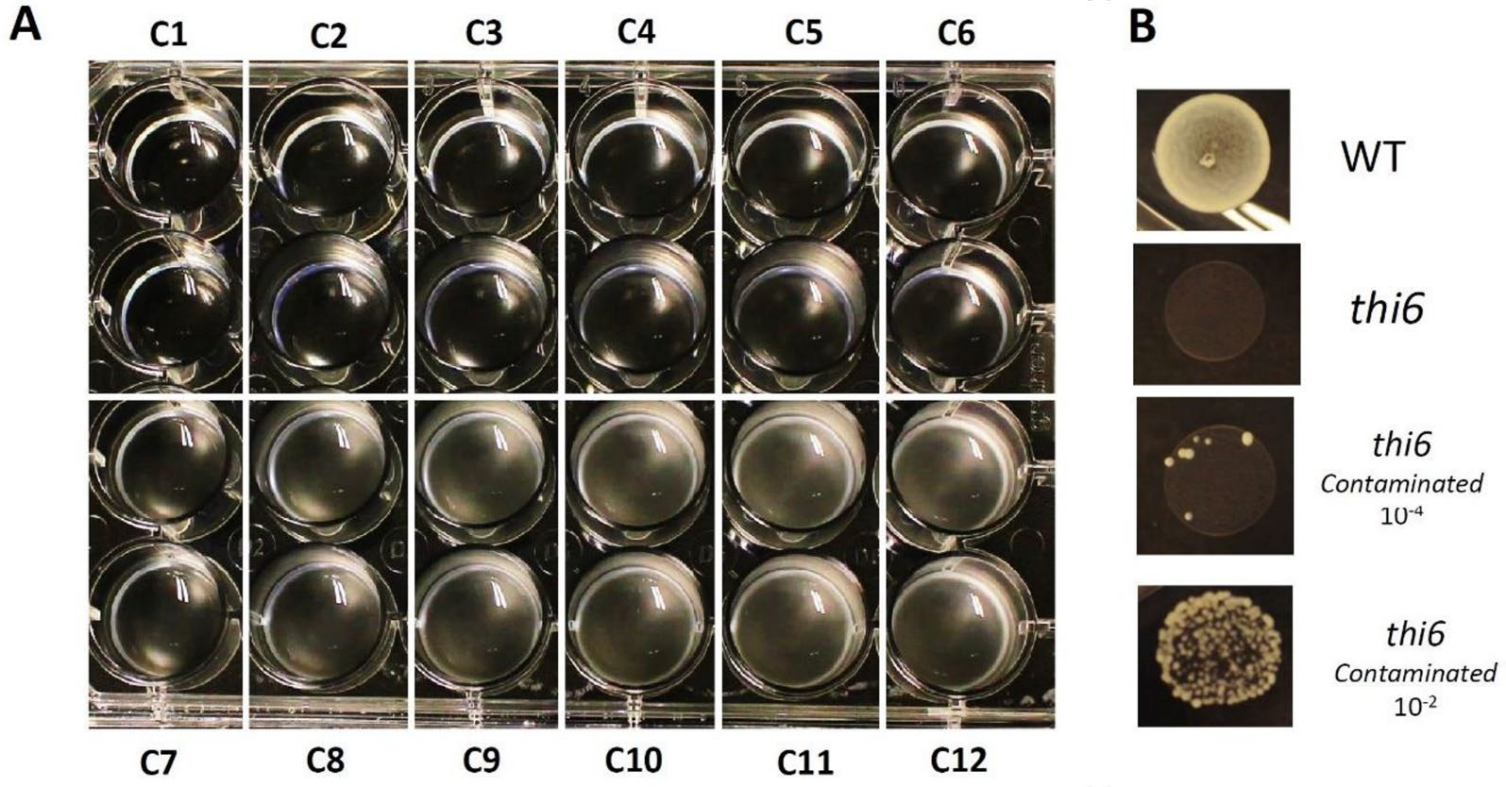
Picture of a standard series 24-well plate and check-up for yeast culture contamination. A 24-well plate used for the assay standard series curve is depicted after 17 h of growth (**A**). In this plate, each standard concentration (C1–C12) is included in duplicate. With the naked eye, increasing turbidity from C1 to C12 is visible. Yeast cultures can be spotted (30 μl) on solidified TFYM (TFYM medium + 1% ultrapure agarose) plates to test for contamination (**B**). WT (Wild Type; BY4741) yeast depicts clear growth on TFYM, while *thi6* shows no growth. Contamination, by inoculating the *thi6* strain with 1/100 (10^–2^) or 1/10.000 (10^–4^) dilution of WT, shows that absence of prototrophic cells can be verified easily

The assay is conducted in a 24-well plate (Greiner), using a 600 µl reaction in each well. This 600 µl reaction volume consists of 250 µl 2xTFYM, 50 µl *thi6* (OD: 0.5) and 233 µl sterile water (which are combined and added to the wells as 533 µl of master mix) and 67 µl sample or standard. For each analysis day, the standard, consisting of multiple (2–4) technical repeats of all 12 standard concentrations, needs to be included. The OD (600 nm) of all wells is measured after 17 h of growth, thereby limiting time differences between measurements of standards and samples. An example of a 24-well plate containing a standard series is presented in Fig. 7A. OD measurement can be done by spectrophotometry (measurement of optical density at 600 nm) or using an automated system such as a plate reader. Here, a TECAN Infinite 200 pro plate reader is used to acquire OD data. Plotting the average observed OD for all specific standard concentrations allows the generation of a (logarithmic) concentration–response curve [28]. By using the logarithmic equation describing the correlation between the observed OD and the vitB_1_ concentration of the standard, the vitB_1_ content of the samples is calculated. Taking a standard series concentration-growth curve in Fig. 1B as an example, the thiamin concentration of an unknown extract is derived using the following equation: concentration (nM) = e^(OD+0.2248)/0.0658^. Importantly, this measurement should be expressed as molar thiamin equivalent (MTE), as it represents the molar quantity of multiple vitB_1_ vitamers (thiamin, TMP, TPP), as compared to a standard series with known thiamin content [28]. The vitB_1_ content of the original plant samples is thereafter deduced from the amount of material used for the extraction.

### Point-by-point protocol

#### Preparation of plant tissue samples

- Precisely weigh 20–200 mg of fresh plant tissue material in a 2 ml tube
  - If this knowledge is available, aim at utilizing material containing 100 to 500 pmol of vitB_1_. Given that the samples are homogenized in 1.74 ml of solvent (see further steps), the vitB_1_ concentrations in the extracts will be in the range of the C2 to C5 standards, for 100 to 500 pmol vitB_1_ starting material, respectively. Utilizing Arabidopsis leaf material as an example, which contains around 2 nmol/g vitB_1_ [30], 100 mg sample weight can be used.
  - In the case of liquid starting material, dilutions in water can be made to match the expected 50–200 nM concentration (in range of standards C2 to C5).
  - Weights should be accurately listed, as the result of the assay needs to be corrected for the amount of starting material.
  - Samples should be flash frozen to be used immediately or to be stored at − 80 °C
- Homogenize the samples
  - Plant tissue samples can be homogenized by addition of glass or metal beads followed by vigorous shaking in specific homogenization equipment, while keeping the samples frozen.
  - Alternatively, mortar and pestle can be used, though this should precede the weighing step, as collection of the complete powdered material is difficult.
- Add 1.5 ml of 22 mM of sulfuric acid (H_2_SO_4_) to homogenized samples
  - Thiamin is more stable in acidic conditions (pH ≈1.5).
- Incubate the acidified samples at 95 °C for 60 min
  - For safety and to prevent sample losses, seal the tubes with parafilm.
  - Note that this extraction step also serves as the sterilization. In all subsequent steps, any contamination should avoided.
- Add 240 µl of 3 M sodium acetate (C_2_H_3_NaO_2_)
  - This step ensures neutralization to a pH of 5.7.
- Centrifuge samples, 5 min at 14,000*g* (4 °C)
  - This step ensures adequate retrieval of clear extracts, as residual turbidity can impact the turbidimetric assay.
- Transfer the supernatant to a sterile 2 ml tube
  - These samples can be used immediately and/or be stored at − 80 °C.
  - Each extract provides sufficient material for over 20 assay reactions.

#### Preparation of the thiamin standard series

- Make a sterile 100 µg/ml thiamin stock solution (vitB_1_ stock)
  - This can be achieved by adding 127 mg of thiamin hydrochloride in 1 L of distilled H_2_O, given the ratio of the molecular weights of thiamin and its hydrochloride form, in which it is typically purchased (thiamin MW: 265.3; thiamin hydrochloride MW: 337.3 g/mol), followed by filter or heat sterilization.
  - This is a 377 µM stock solution, used to acquire the thiamin dilution series.
- Make thiamin concentrations C1–C12, using the 377 µM stock solution (100 µg/ml)
  - The concentrations are as follows: C1, 0 nM; C2, 47 nM; C3, 94 nM; C4, 141 nM; C5, 188 nM; C6, 188 nM; C7, 329 nM; C8, 471 nM; C9, 707 nM; C10, 942 nM; C11, 1885 nM; C12, 3770 nM.
  - The concentrations for all standards are made by diluting the following amounts of the 377 µM thiamin stock solution (100 µg/ml) in sterile H_2_O up to 40 ml: C1, 0 µl; C2, 5 µl; C3, 10 µl; C4, 15 µl; C5, 20 µl; C6, 25 µl; C7, 35 µl; C8, 50 µl; C9, 75 µl; C10, 100 µl; C11, 200 µl; C12, 400 µl.
  - Solutions should be stored and − 20 °C and can be used multiple times

#### Preparation of the yeast culture

- Inoculate YPD plate (containing vitB_1_) with yeast strain (*thi6*), to allow single colony growth
  - The presence of vitB_1_ in this complex growth medium ensures growth of the auxotrophic yeast strain.
- Inoculate 50 ml liquid TFYM, supplemented with thiamin (100 ng/ml) and cultivate until growth is visible (OD 0.5–1.5; 16 h at 28 °C, 200 rpm)
  - The same TFYM medium as will be used in the assay is utilized here, with the exception that it is supplemented with 377 nM (100 ng/ml; 1000× dilution of the 377 µM solution, see ‘preparation of the thiamin standard series’).
  - The 50 ml culture should support a sufficient amount of yeast material for over 1000 assay reactions, as 50 µl of 0.5 OD is used per assay (see ‘preparation of the assay’).
- Collect yeast cell pellet by centrifugation (1700*g*, 5 min)
  - The forces applied should be non-destructive, as the living cells are needed for the downstream assay.
- Gently resuspend yeast cell pellet in 0.85% NaCl (sterile!) and pellet down (1700*g*, 5 min)
  - This washing step is included to eliminate the presence of vitB_1_ (needed for growth), while limiting osmotic shock to the cells.
- Repeat washing step 3 times.
- Resolve pellet in 10 ml TFYM (no vitB_1_!).
- Dilute to an OD of 0.5
  - Checking whether the culture is contamination-free can be done by growth on solidified TFYM plates (TFYM+ 1% ultrapure agarose) (Fig. 7B).

#### Preparation of the assay

- Create a ‘mastermix’ for 100 assay reactions: 25 ml 2xTFYM, 5 ml yeast culture at OD 0.5, 23.3 ml water (sterile!)
  - This mastermix can be used for the complete assay. Here the volume for 100 reactions is given as an example.
- Pipet 533 µl mastermix in all assay recipients (e.g. wells of a 24-well plate).
- Reserve 36 reactions for the thiamin standards series, in which 67 µl of standard is added to 533 µl of mastermix, 3 times for each of the 12 standards (see ‘Preparation of the thiamin standard series‘).
- Add 67 µl of thiamin extract to 533 µl of mastermix for analysis of the samples
  - Include a sufficient number of technical (N ≥ 2) and biological (N ≥ 3) replicates for each sample.
- Grow the cultures for 17 h (28 °C, 200 rpm) and measure OD values for all assay reactions
  - If multiple plates or cultures need to be measured, a portion can be stored at 4 °C to limit further growth.
- Derive the equation, describing the relationship between thiamin concentration and measured OD, by using the averages of the OD’s measured for each standard as a relation to its known concentration.
- Utilize the derived equation to deduce the vitB_1_ concentration in the samples
  - Use the means of OD measured in technical replicates for each concentration.
- Apply the equation to calculate the vitB_1_ concentration of the plant tissue extracts.
- With the derived vitB_1_ concentration of the extracts, the vitB_1_ content of the plant tissue samples can be obtained, taking the original weight of the sample into account.
  - The sample (weight = X; in gram) was diluted in a 2 ml tube containing 1.74 ml liquid (1.5 ml + 240 µl), the dilution (or concentration difference between extract and plant tissue) can then be approximated as X/(1.74 + X). Note that this approximation presumes a density of 1 ml/g for the samples, which can deviate for samples with lower water content. However, as sample weight is kept below 200 mg, this deviation has limited effect on the estimation of the dilution.
- Utilizing the means of different biological repeats (e.g. multiple extractions from different plants of a particular line/cultivar of interest), the vitB_1_ content of these different lines/cultivars can be compared.
  - To make data more trustworthy, or to verify results, the complete assay can be repeated (another day). Keep in mind that each assay should be accompanied by a new standard series.

### Materials and equipment

#### Chemicals

- Salt, NaCl; sulfuric acid, H_2_SO_4_; sodium acetate, C_2_H_3_NaO_2_.

#### Media

- Yeast extract-Peptone-Dextrose (YPD) medium, solid:
  - 10 g yeast extract, 20 g bacterial peptone, 15 g agar, add H_2_O to 1 l.
- Thiamin Free Yeast medium (2xTFYM):
  - 6.9 g of yeast nitrogen base without amino acids and without thiamin (CYN4701, ForMedium), 790 mg complete supplement medium (DCS0011, ForMedium), 10 g sucrose is added to 500 ml of water to make 2xTFYM (which is diluted 2× to make 1xTFYM).

### Equipment

- Plate reader (including 24-well plates) or other OD measuring device
  - Tecan Infinite M Plex with Greiner 24-well plates was utilized here
- Microbial culture tubes (1–5 ml)
- 28 °C shaking incubator

## Data Availability

The datasets used and/or analyzed during the current study are available from the corresponding author on reasonable request.

## Abbreviations

MW: Molecular weight
OD: Optical density
RDA: Recommended daily allowance
TFYM: Thiamin-free yeast medium
TMP: Thiamin monophosphate
TPP: Thiamin pyrophosphate
TTP: Thiamin triphosphate
VitB_1_: Vitamin B_1_ (sum of thiamin, TMP and TPP)
YPD: Yeast extract-peptone-dextrose medium
